# Active Learning of Biostatistics in Medical Education: An Educational Intervention Using Algorithms, Article Analysis and SPSS Simulation

**DOI:** 10.7759/cureus.103464

**Published:** 2026-02-12

**Authors:** Néstor Israel Quinapanta Castro, Carlos Escobar, Jorman F Choez-A

**Affiliations:** 1 Department of Doctoral Studies, Faculty of Philosophy and Letters, University of Buenos Aires, Buenos Aires, ARG; 2 Department of Research, Autonomous Regional University of the Andes (UNIANDES), Ambato, ECU; 3 Department of Medicine, Autonomous Regional University of the Andes (UNIANDES), Ambato, ECU; 4 Methodology of Health Sciences Research, International University of La Rioja, La Rioja, ESP

**Keywords:** applied biostatistics, education and training, medical education and training, medical school education, teaching biostatistics

## Abstract

Introduction

Although biostatistics is a core component of medical education, traditional lecture-based teaching methods can limit students' understanding of, and ability to apply, statistical concepts in practice. Active, multimodal pedagogical strategies can improve learning outcomes and student engagement.

Objective

The objective of this study is to evaluate the effect of an active, multimodal pedagogical intervention (algorithms, SPSS simulation and article analysis) on the following among medical students: academic performance, the perceived mastery of course content, importance attributed to parametric and nonparametric tests and satisfaction with the teaching method.

Methods

A parallel-group intervention study was conducted among third-semester medical students enrolled in a biostatistics course between October 2025 and March 2026. The participants were randomly assigned using simple randomisation to either the intervention group (n = 22) or the control group (n = 39). While the control group attended traditional lectures, the intervention group participated in an active, multimodal programme incorporating algorithm-based instruction, the critical analysis of scientific articles and practical SPSS software simulations. Academic performance was assessed using a written biostatistics examination, while perceptions of importance, mastery and satisfaction were measured using Likert scales. Group comparisons were performed using Student's t-test, a Mann-Whitney U test and a chi-square test, with a significance level of p < 0.05.

Results

The intervention group achieved significantly higher examination scores than the control group (13.00 ± 3.84 versus 9.44 ± 4.10, p = 0.001). A greater proportion of students in the intervention group obtained a favourable result (≥14 points). The odds ratio (OR) (OR = 0.107; 95% confidence interval {CI}: 0.013-0.891) reflects the reduced odds of failure in the intervention group, indicating a significant association between group membership and academic success. Additionally, the intervention group reported attributing significantly greater importance to statistical tests (p = 0.036), perceiving themselves as having a significantly greater mastery of the course content (p = 0.017) and being significantly more satisfied with the pedagogical method (p = 0.015).

Conclusion

The active, multimodal pedagogical intervention had a significant positive effect on academic performance and students' perceptions of learning in biostatistics. These findings support the incorporation of algorithm-based instruction, the critical appraisal of scientific literature and statistical software simulation as effective strategies to enhance biostatistics education in medical training.

## Introduction

Inferential statistics enable conclusions to be drawn about a population based on a sample, primarily through parameter estimation and hypothesis testing [1]. Statistical tests establish the probability that a conclusion obtained from a sample is applicable to the population from which it was obtained [2]. Choosing the right statistical method is important to avoid making mistakes and depends on the objectives, hypotheses, type of study, sample size and sampling method, measurement scale and whether the groups are independent or paired [3].

Parametric tests are based on assumptions about the parameters of the data distribution. They generally assume a normal distribution, considering factors such as the mean and standard deviation (SD) [4]. Nonparametric statistics, on the other hand, are used when the data do not meet the assumptions of parametric tests or when the variables are measured on nominal or ordinal scales [5]. Nonparametric techniques are preferable when the data are highly skewed with severe outliers (where the median better represents the data than the mean) or when the sample size is very small, making normality difficult to assess [6].

The t-test and ANOVA make several assumptions, including normality, independence and equal variances. In contrast, their nonparametric alternatives (Mann-Whitney U, Wilcoxon, Kruskal-Wallis, chi-square and Fisher's exact tests) require less stringent assumptions [7,8]. When the data come from a normal distribution, the t-test has greater statistical power than the Mann-Whitney test. However, when the data come from a variety of non-normal distributions, the Mann-Whitney test is superior [9]. The Kruskal-Wallis test is used for more than two independent groups: the Wilcoxon signed-rank test for paired data, the Friedman test for repeated measures and Spearman's correlation to analyse associations between quantitative and ordinal variables [10].

However, choosing the appropriate statistical test can be challenging for novice researchers, who often struggle to apply theoretical statistical concepts to practical situations. This can hinder their ability to apply this knowledge in future careers [11]. In basic biomedical research, errors relating to the misuse of t-tests, ANOVA, repeated measures analysis, nonparametric tests and multiple comparisons are common [12].

Given its key role in the critical interpretation of scientific evidence and clinical decision-making, training in biostatistics is a fundamental component of the medical degree curriculum. Several studies have emphasised the importance of this training going beyond theoretical content to include the balanced integration of practical skills applicable to the clinical context. In this regard, a survey of 278 medical graduates who teach or have taught medical students showed that the majority consider it essential for statistics teaching to combine conceptual understanding with practical skills, particularly for topics directly related to medical practice [13].

Similarly, a significant improvement in knowledge levels was observed in a medical student educational intervention comprising 20 hours of theory and 12 hours of practice. Before the course, 68.0% of the students reported having basic knowledge of biostatistics, achieving an average score of 2.5 ± 1.4. After training, this proportion increased to 95.7%, achieving an average score of 7.5 ± 2.1 (p < 0.001). Furthermore, the understanding of the key content (population and sample, measures of central tendency and dispersion, hypothesis formulation, parametric and nonparametric tests and the use of SPSS) increased from below 71% before training to over 93% after training [14].

The primary objective was to evaluate the effect of an active pedagogical intervention focusing on algorithms, the critical analysis of scientific articles and SPSS simulations on the academic performance of university students enrolled on a biostatistics course compared to traditional teaching methods. The secondary objectives were to assess the impact of the intervention on students' perception of content mastery and the importance they attributed to statistical tests, as well as their satisfaction with the pedagogical method.

## Materials and methods

Study design

An educational intervention was carried out with equivalent parallel groups consisting of an intervention group and a control group. Two hundred twelve third-semester medical students enrolled on the biostatistics course were randomised.

Sample size

**Figure 1:**
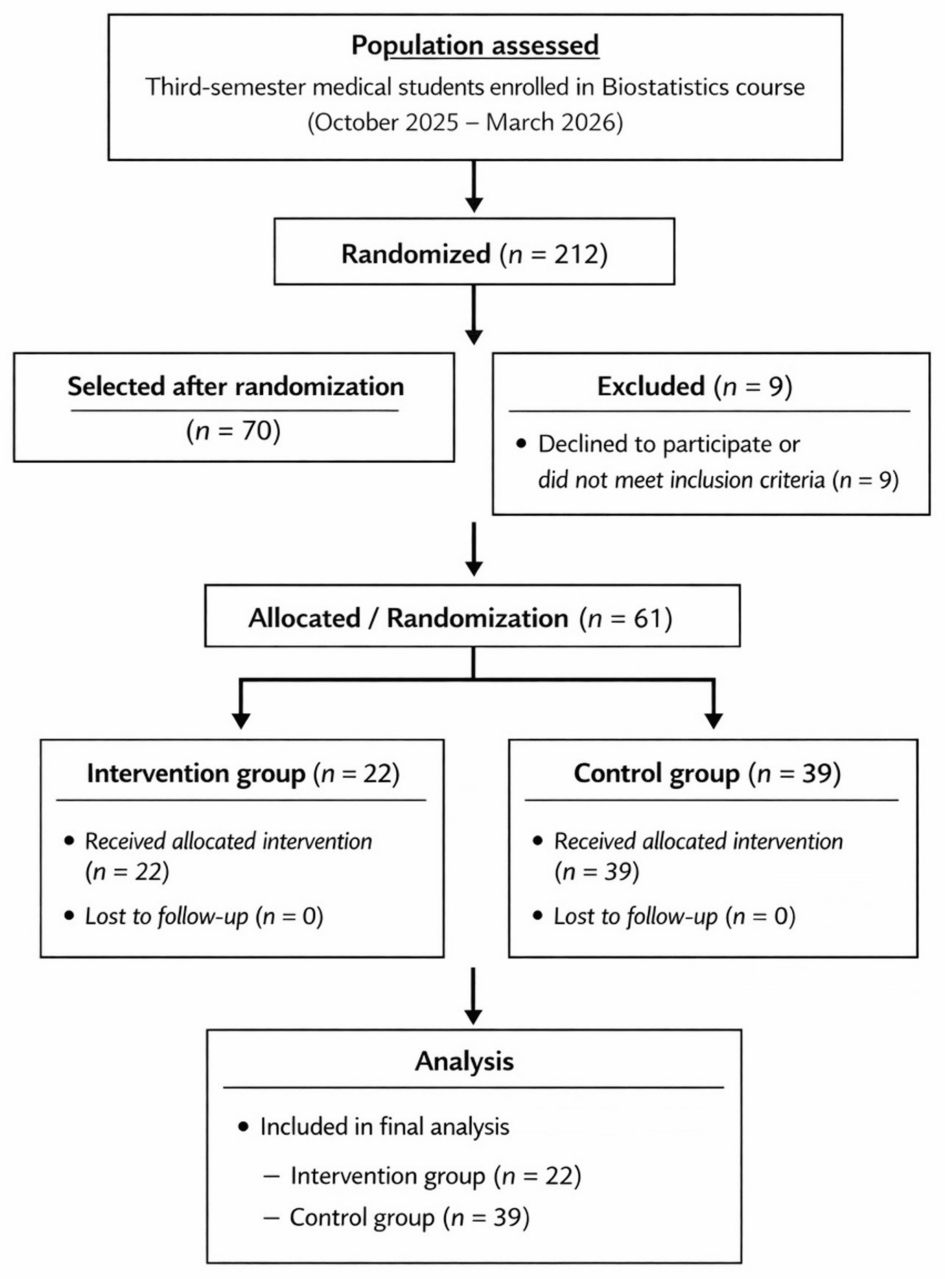
Student selection flowchart

The study population consisted of third-semester medical students enrolled on the biostatistics course between October 2025 and March 2026. The participants were selected through simple random sampling and randomly assigned to the intervention or control group in a blinded manner using SPSS software version 29 (IBM Corp., Armonk, NY). Of these, 22 were assigned to the intervention group and 39 to the control group (see Figure 1). There were no losses during the follow-up period for either group, and all participants assigned to the study were included in the final analysis.

Only students who were officially enrolled on the Research and Biostatistics I course, had attended at least 80% of academic sessions and had signed the informed consent form to participate in the study were included. Students who did not complete the academic evaluation, did not attend 100% of training sessions or provided incomplete information in the data collection instruments were excluded.

Intervention

**Figure 2:**
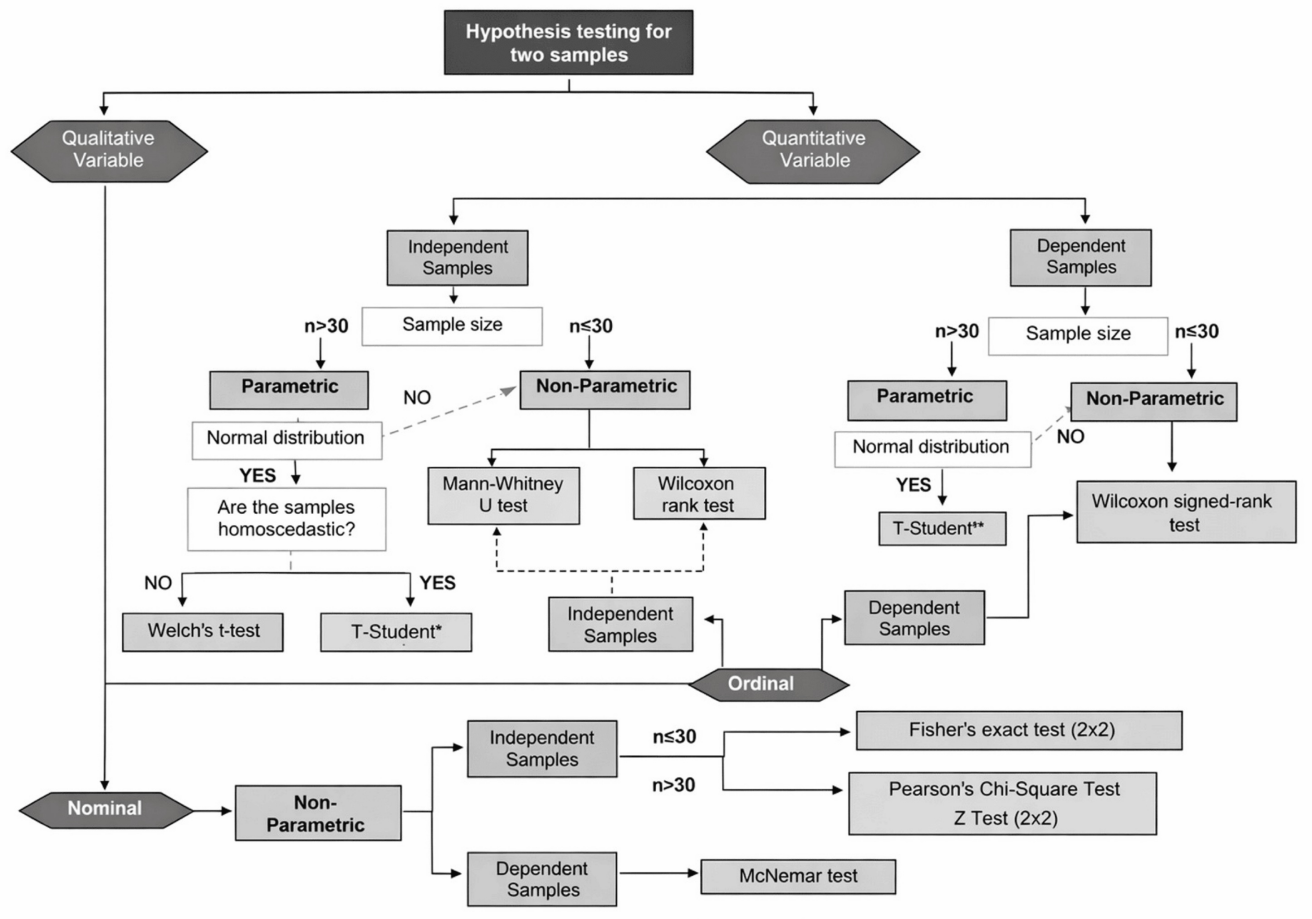
Algorithm: hypothesis testing for two samples *Independent samples t-test **Paired sample t-test

**Figure 3:**
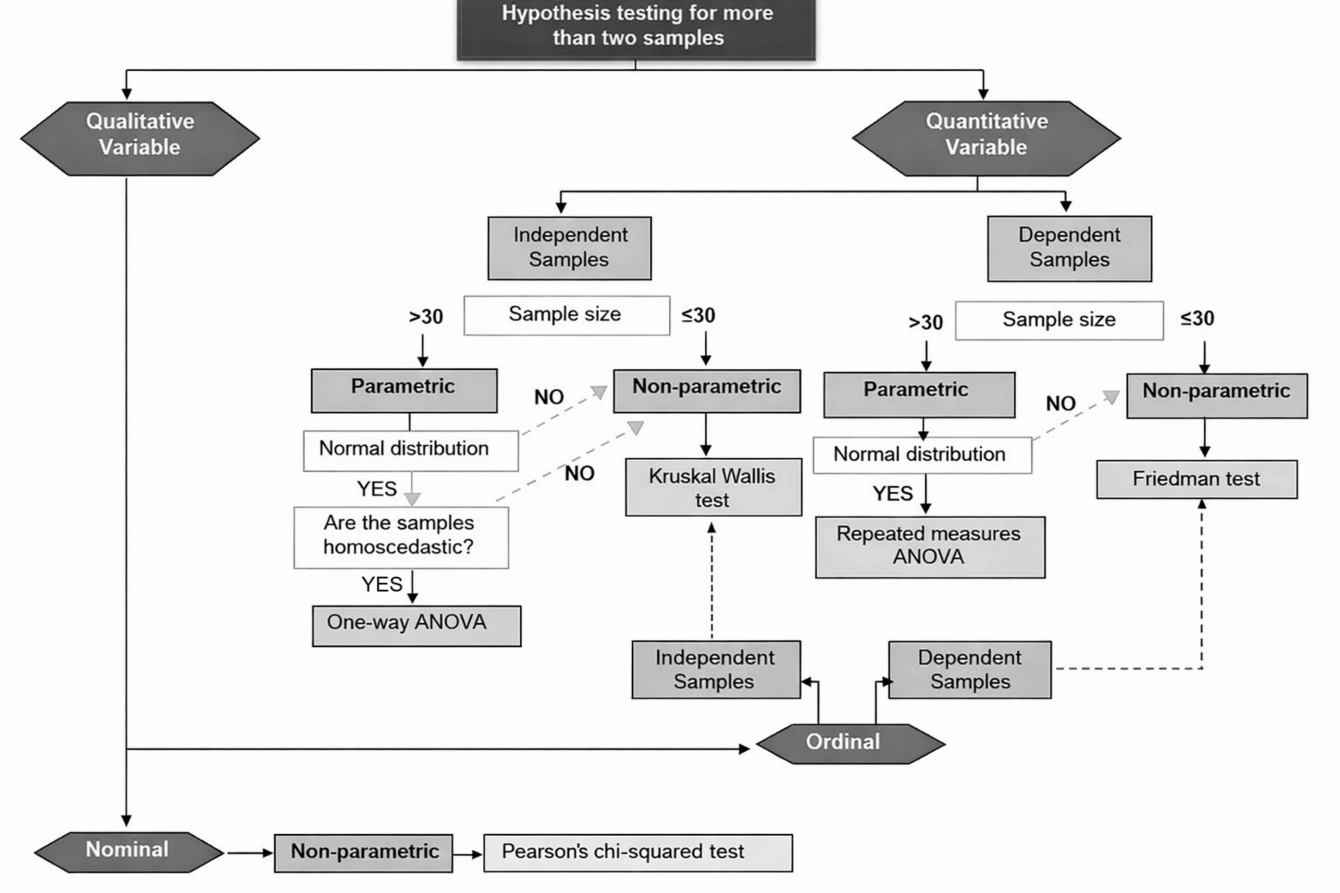
Algorithm: hypothesis testing for more than two samples

The control group received traditional teaching methodology based on conventional lectures focusing on the theoretical explanation of biostatistics content, accompanied by examples and exercises solved by the teacher. The intervention group was exposed to an active, multimodal pedagogical strategy structured into three integrated components. Firstly, an algorithm-based instructional session was implemented to encourage structured statistical thinking. These algorithms were designed as step-by-step guides to support the process of hypothesis testing. This includes identifying the study design, classifying the variables, assessing the assumptions about the distribution of the data, selecting parametric or nonparametric tests, executing the analysis and interpreting the results. Two algorithms were employed: one for hypothesis testing with two samples (Figure 2) and another for comparisons with more than two samples (Figure 3). These algorithms were applied through guided problem-solving exercises based on clinical and epidemiological scenarios.

Secondly, the students performed a guided critical appraisal of scientific articles. During this activity, they identified the study design and types of variables and evaluated the coherence between the objectives, methods, results and conclusions. The aim of this component was to reinforce the practical application of statistical concepts and enhance the interpretation of biostatistical analyses in real research contexts.

Thirdly, practical simulation activities were conducted using SPSS statistical software. Working with predefined databases, students applied the learned algorithms, performed descriptive and inferential statistical analyses, executed appropriate hypothesis tests and interpreted statistical outputs (e.g. p-values, confidence intervals and test statistics). These exercises were conducted under instructor supervision with step-by-step guidance.

The activities of the intervention group were carried out in three face-to-face sessions, comprising two theoretical and practical sessions (lectures based on algorithms and the interpretation of scientific articles), as well as a three-hour simulation session using the SPSS database. The total duration of the teaching course was seven hours in both the intervention and control groups, ensuring comparable academic conditions between groups. The control group received conventional instruction over the same total instructional time. Instructional delivery in both groups was supported by the use of interactive digital whiteboards (ViewBoard® IFP9851, ViewSonic, Brea, CA).

Knowledge acquisition was assessed using a biostatistics knowledge test consisting of 20 questions (Appendices). The examination was scored on a scale from zero to 20 points, with one point awarded for each correct answer and higher scores indicating better performance.

Data collection instruments

Information was collected using a set of structured instruments. Primarily, a sociodemographic and academic questionnaire was administered, including variables such as gender, age, academic semester, current living situation, number of semesters lost, number of semesters repeated, ethnicity and geographical origin.

An objective academic assessment was also used to measure student performance. This was expressed as a total score obtained in a written biostatistics test designed to assess theoretical knowledge and the ability to interpret statistical tests and apply concepts. The test comprised 20 questions similar to those found in the Medical Intern Resident (MIR) examination. The content validity of this instrument was evaluated by three experts in biostatistics and research methodology, including a medical epidemiologist, the head of the research department and two research professors from the medical school. The instrument was also formally validated and approved by the director of the medical programme.

Likert scales were also applied to assess the importance attributed to parametric and nonparametric tests (scale of 1-7), perceived mastery of class content (scale of 1-10) and satisfaction with the teaching method used (scale of 1-5).

Procedure

In the first stage, the participants were asked to provide their sociodemographic and academic information. Academic sessions were then developed for each group, applying the assigned pedagogical methodology. Following the intervention, both groups were evaluated under equivalent conditions using the same objective academic test, as well as perception and satisfaction instruments.

The algorithms, instructional sessions and assessment questions were developed collaboratively by faculty members from the biostatistics department through a peer-review process. The head of the research department reviewed and supervised all educational materials, including the algorithm-based classes, SPSS simulation activities and the biostatistics knowledge test, to ensure academic coherence, methodological rigour and alignment with the official curriculum.

The aim of this peer-based development and supervisory process was to standardise instructional content, reduce individual instructor bias and ensure consistency across teaching sessions and assessment instruments.

Statistical analysis

Data analysis was performed using SPSS version 29. Descriptive analysis was performed using absolute and relative frequencies for categorical variables and means and standard deviations for continuous variables. Comparisons between groups were made using Student's t-test for independent samples for continuous variables and a Pearson's chi-square test for categorical variables. Additionally, 95% confidence intervals (CI) for the differences in means and odds ratios (OR) with their respective confidence intervals were calculated for the analysis of dichotomous variables. A binary logistic regression analysis was performed to identify factors associated with the outcome variable. The independent variables included group assignment (intervention versus control), ethnicity, sex and history of academic semester loss. A statistical significance level of p < 0.05 was established.

Ethical considerations

The study was conducted in accordance with the ethical principles governing research involving human subjects. Participation was voluntary, and informed consent was obtained from all students. All information collected was treated confidentially and anonymously and was used exclusively for academic and scientific purposes. Approval was granted internally by the career directorate and the head of research. As the research formed part of an institutional educational evaluation and posed no risks to the participants, approval by an external ethics committee was not required.

## Results

The sample consisted of 61 participants with a mean age of 20.36 years (SD = 1.95). In terms of gender, 33 participants were men (54.1%), and 28 were women (45.9%). In terms of current living arrangements, 33 participants (54.1%) lived with their parents, 21 (34.4%) lived alone, two (3.3%) lived with their grandparents, four (6.6%) lived with other relatives and one (1.6%) lived elsewhere. Academically, 42 students (68.9%) had not lost any semesters, while 19 (31.1%) had lost at least one. In terms of ethnic self-identification, 57 participants (93.4%) identified as mestizo, while four (6.6%) identified as indigenous. Geographical origin showed a marked predominance of the Sierra region (54 students, 88.5%), followed by the Amazon region (five students, 8.2%) and the Coast (two students, 3.3%).

No statistically significant differences were identified between the two groups in any of the analysed variables (p > 0.05) in Table 1. The groups were comparable in terms of gender, academic semester, cohabitation, semester loss, ethnicity and geographical origin, indicating sociodemographic and academic homogeneity.

**Table 1:** Baseline characteristics of the participants

Variable	Category	Intervention, n (%) (n = 22)	Control, n (%) (n = 39)	χ²	p
Sex	Female	10 (0.164)	18 (0.295)	0.003	0.958
	Male	12 (0.197)	21 (0.344)		
Living arrangement	Living alone	10 (0.164)	11 (0.180)	6.644	0.156
	Living with parents	9 (0.148)	24 (0.393)		
	Living with grandparents	0 (0.000)	2 (0.033)		
	Living with relatives	3 (0.049)	1 (0.016)		
	Other living arrangements	0 (0.000)	1 (0.016)		
Academic semester loss	No semester loss	17 (0.279)	25 (0.410)	1.138	0.286
	Semester loss	5 (0.082)	14 (0.230)		
Ethnicity	Indigenous	1 (0.016)	3 (0.049)	0.227	0.634
	Mestizo	21 (0.344)	36 (0.590)		
Geographical origin	Highlands (Sierra)	19 (0.311)	35 (0.574)	4.123	0.127
	Amazon region	1 (0.016)	4 (0.066)		
	Coastal region	2 (0.033)	0 (0.000)		

The analysis of the means showed no statistically significant differences between the groups in terms of participant age (t = 0.690; p = 0.493), with a mean difference of 0.360 years (95% CI: -0.685 to 1.405). Similarly, no significant differences were observed in the number of semesters repeated (t = -0.774; p = 0.442), with a mean difference of -0.143 (95% CI: -0.514 to 0.227). These results suggest that the two groups were comparable in terms of age and academic background.

Total score

The analysis comparing means revealed statistically significant differences between the groups for all evaluated variables (Table 2). Taken together, these findings suggest a positive differential effect in favour of the group with higher means in the evaluated variables.

**Table 2:** Comparison of mean scores between the intervention and control groups *Mann-Whitney U test **Student's t-test SD, standard deviation; IQR, interquartile range; t, Student's t-test; U, Mann-Whitney U test; CI, confidence interval

Variables	Intervention (mean ± SD/median {IQR})	Control (mean ± SD/median {IQR})	t/U	p	Mean difference	95% CI, lower	95% CI, upper
Examination score (0-20)	13.00 ± 3.84	9.44 ± 4.10	3.394	0.001**	3.564	1.451	5.678
Importance of parametric and nonparametric tests (1-7)	7.00 (1)	6.00 (2)	304.000	0.036*			
Mastery of course content (1-10)	7.50 (3)	6.00 (2)	272.500	0.017*			
Satisfaction with the pedagogical method (1-5)	5.00 (1)	4.00 (2)	283.000	0.015*			

The mean score for the intervention group was 13.00 ± 3.84, while the control group had a mean score of 9.44 ± 4.10. The Shapiro-Wilk test showed that the data followed a normal distribution in both groups (p > 0.05). Similarly, Levene's test revealed the homogeneity of variances (F = 0.786; p = 0.380), thus assuming the equality of variances for the contrast. There was a statistically significant difference in the means of the two groups (t = 3.394; p = 0.001), with an average difference of 3.564 points (95% CI: 1.451-5.678). This indicates that the intervention had a favourable effect.

**Figure 4:**
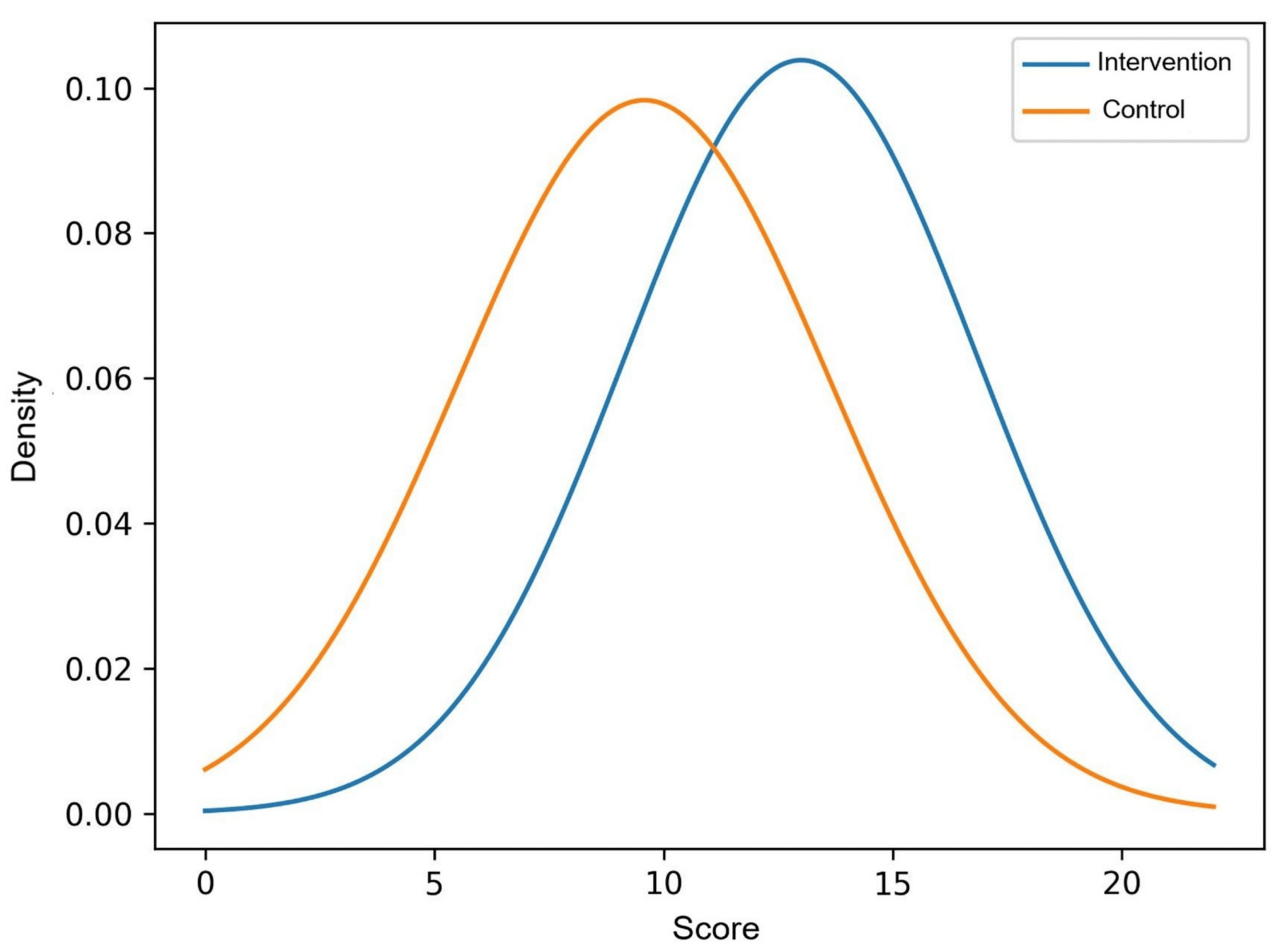
Examination score distribution by the intervention (n = 22) and control (n = 39) groups

Figure 4 showed that the intervention group achieved higher average scores than the control group, with similar variability in both groups. Overall, the intervention group performed better.

The examination score was previously converted into a dichotomous variable, with a score of 14 points or higher considered favourable (approved). In a sample of 61 participants, a statistically significant association was found between group (intervention versus control) and achieving 14 points or more in the examination (Pearson's χ² = 5.77; p = 0.016). The participants in the intervention group were significantly less likely to obtain an unfavourable result (score < 14) than those in the control group (OR = 0.107; 95% CI: 0.013-0.891).

The logistic regression model indicates that the probability of passing or failing is significantly influenced by belonging to the intervention or control group (p = 0.007), while variables such as ethnicity, gender or semesters missed were not significant. The model explains between 13% and 18% of the variability in the results and has an overall accuracy of 73.8%, better predicting students who do not pass (80.5%) than those who do pass (60%), suggesting that the intervention has a moderate but relevant effect on academic performance (Table 3).

**Table 3:** Factors associated with the outcome: logistic regression results df: degrees of freedom

Variables	B	Standard error	Wald	df	Significance	Exp(B)
Group	-1.566	0.601	6.785	1	0.009	0.209
Ethnicity identified	1.098	1.158	0.898	1	0.343	2.998
Have you lost any academic semester?	-0.381	0.675	0.318	1	0.573	0.683
Sex	-0.278	0.610	0.207	1	0.649	0.758
Constant	1.946	1.036	3.533	1	0.060	7.004

Importance attributed

**Figure 5:**
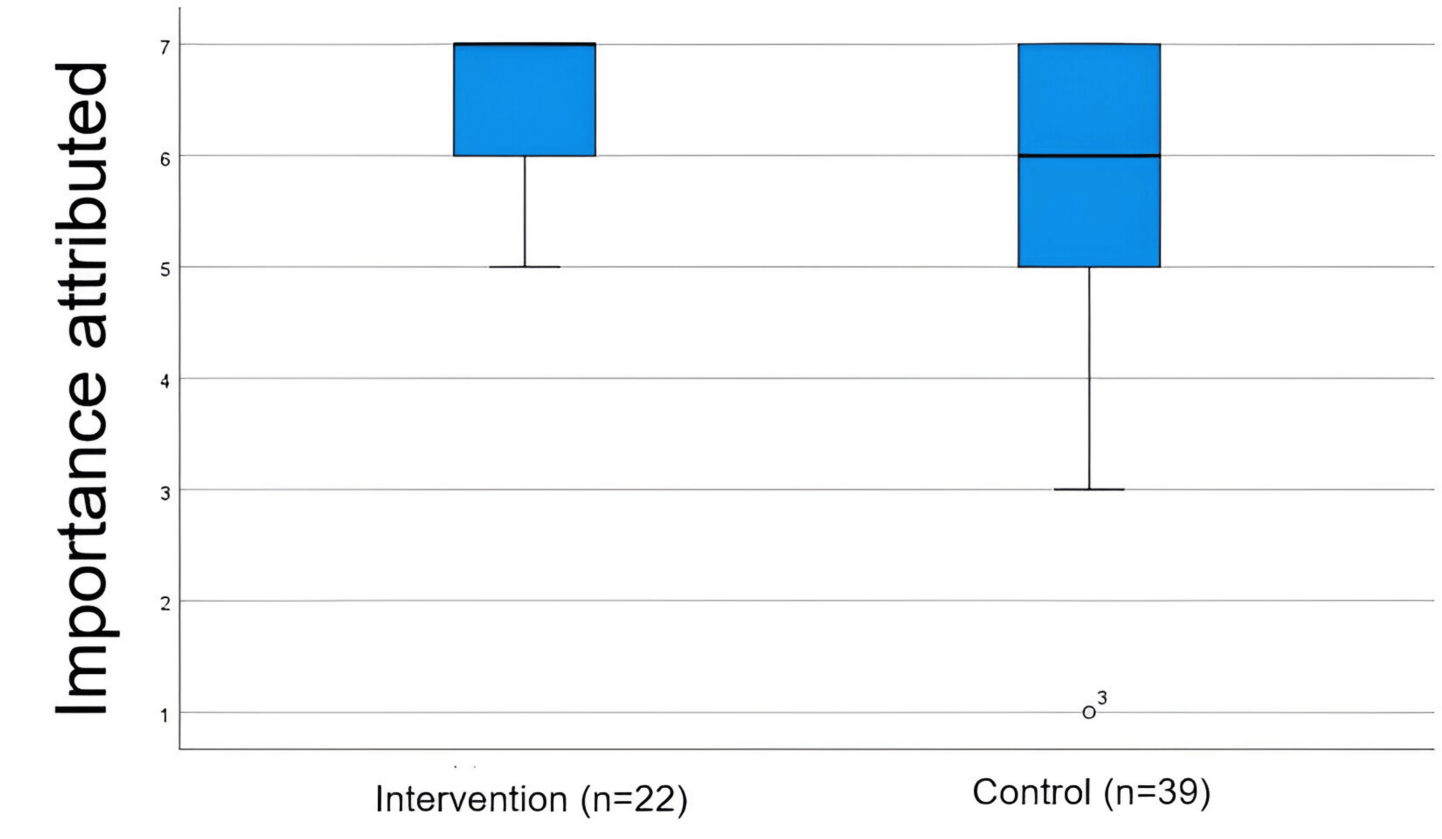
Importance attributed to the application of parametric and nonparametric tests in statistical training, by group

The intervention group placed greater importance on applying parametric and nonparametric tests (median = 7; IQR = 1) than the control group (median = 6; IQR = 2), and this difference was statistically significant (Mann-Whitney U = 304; p = 0.036), as shown in Table 2. The box plot shows that both groups consider the application of parametric and nonparametric tests to be important for their training, as indicated by the high medians close to the upper values of the scale (Figure 5). However, the intervention group shows more consistent responses. In contrast, the control group is more heterogeneous and records lower values, including extreme cases.

Perceived mastery

**Figure 6:**
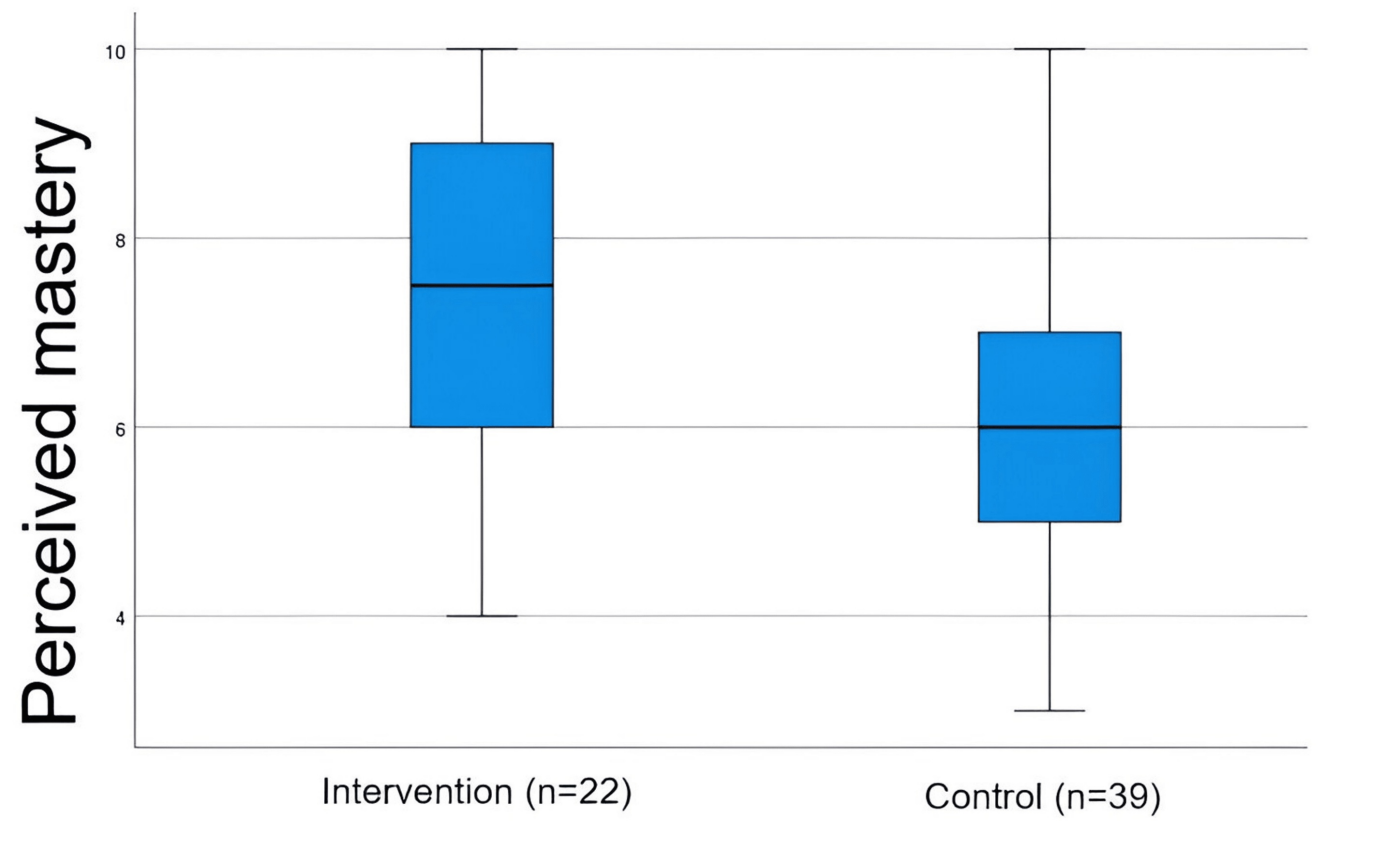
Perceived mastery by groups

The intervention group reported greater perceived mastery of class content (median = 7.5; IQR = 3) than the control group (median = 6; IQR = 2), and this difference was statistically significant (Mann-Whitney U = 272.5; p = 0.017), as shown in Table 2. The box plot shows that compared to the control group, the intervention group reports greater perceived mastery of the concepts and content of the class, with a higher median and a distribution centred on higher values on the scale (Figure 6). The control group's median is lower and shows greater dispersion towards lower values.

Satisfaction

**Figure 7:**
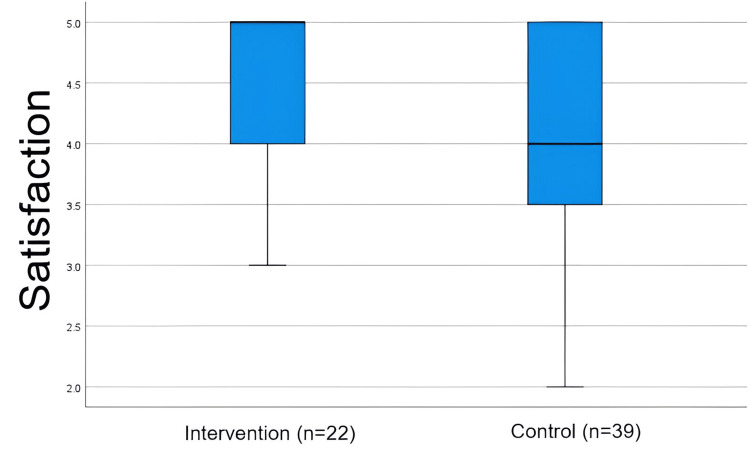
Satisfaction by each group

The intervention group reported higher satisfaction with the teaching method (median = 5; IQR = 1) than the control group (median = 4; IQR = 2), and this difference was statistically significant (Mann-Whitney U = 283; p = 0.015), as shown in Table 2. As can be seen from the box plot (Figure 7), both groups attach high importance to it, but the intervention group is more homogeneous and has higher scores, while the control group shows greater variability and lower values.

## Discussion

No initial differences were observed between the intervention group and the control group in terms of sociodemographic or academic characteristics in 61 medical students. Following the intervention, however, the intervention group achieved better academic results, reported a greater sense of mastery of the content, rated statistical tests more highly and expressed greater satisfaction with the teaching method (p < 0.05). Furthermore, belonging to the intervention group was significantly associated with a higher probability of achieving high grades, indicating the intervention's positive impact.

The results of this study are consistent with previous evidence that highlights the superiority of active, practical and student-centred pedagogical approaches to traditional lecture-based methods for teaching biostatistics in medicine [15-20]. Overall, these studies agree that educational models integrating practical application, interaction and clinical contextualisation promote a better understanding of, and greater confidence in, biostatistics and lead to better learning outcomes.

Quinapanta Castro and Orbea evaluated a 'three-in-one' active teaching approach, combining article analysis, SPSS simulations and lectures, for medical students [15]. In their study, the intervention group achieved significantly higher academic performance, reporting greater satisfaction and a better perceived understanding than students who received traditional instruction. Similarly, our results support the idea that integrating theoretical content with applied, contextualised activities improves student engagement and facilitates a deeper understanding of biostatistics.

In this context, Hayes et al. demonstrated that an educational module integrating conceptual definitions, examples from medical literature and formative assessments with immediate feedback was associated with significant improvements in objective knowledge and confidence in selecting and interpreting statistical tests [16]. Consistent with this, studies comparing problem-based learning (PBL) with traditional teaching methods revealed superior academic performance and a heightened sense of usefulness, critical thinking and self-directed learning among the PBL group [17]. Similarly, the biostatistics literacy course at the University of Minnesota demonstrated the effectiveness of an approach focused on the statistical reasoning and critical reading of scientific articles rather than mechanical calculation on a large scale and in various teaching formats [18].

Similarly, a Polish study [19] found that practical biostatistics teaching was associated with lower academic stress, greater satisfaction and a better perception of applied skill acquisition, while a Brazilian study [20] conducted in 2017 reported that incorporating R and RStudio into biostatistics teaching was associated with a more favourable attitude towards statistics among students, greater perceived competence and better academic performance.

Taken together, these results suggest that technology-supported, practice-oriented instructional approaches can improve cognitive and emotional learning outcomes in biostatistics. However, the ROME programme in India emphasised the importance of practical and systematic training in biostatistics for clinical practice and future academic development [21].

Limitations

Firstly, the sample size was small, consisting of students from a single institution and subject area, which limits the generalisability of the results to other educational contexts. Secondly, several of the analysed variables were based on students' self-perceptions, which may have introduced social desirability bias or an overestimation of the intervention's impact. Furthermore, the evaluation was only conducted in the short term without any longitudinal follow-up, making it impossible to determine whether the effects would be stable over time.

## Conclusions

This study demonstrates that an active, multimodal pedagogical intervention incorporating algorithm-based instruction, the critical analysis of scientific articles and practical simulations using statistical software positively impacts biostatistics learning outcomes among medical students. Students who received the intervention performed significantly better academically than those who received traditional lecture-based instruction, in terms of both mean examination scores and the likelihood of achieving a favourable performance threshold.

In addition to improving objective academic performance, the intervention was also associated with significant improvements in students' perceptions of the learning process. The participants in the intervention group placed greater value on the application of parametric and nonparametric statistical tests, reported a higher level of mastery of the course content and were more satisfied with the teaching method than those in the control group. These findings suggest that the intervention enhanced not only knowledge acquisition but also statistical reasoning, confidence and engagement with biostatistical concepts.

## Appendices

Table 4 shows the biostatistics knowledge test consisting of 20 questions.

**Table 4:** Original questionnaire developed for academic purposes in biostatistics Credits: Quinapanta Castro NI and Escobar C. Note: correct answers are indicated in bold

Number	Questions	Answer options	Questions	Answer options
1	¿Cuál de las siguientes condiciones es necesaria para aplicar una prueba paramétrica?	A) Tamaño muestral pequeño. B) Datos en escala nominal. C) Distribución normal de los datos.D) Variables cualitativas	Which of the following conditions is required to apply a parametric test?	A) Small sample size. B) Nominal scale data. C) Normal distribution of the data. D) Qualitative variables
2	Un investigador analiza los niveles de cortisol (mg/dL) en sangre en 190 diabéticos y no diabéticos. Los datos siguen una distribución normal y las varianzas son homogéneas. ¿Qué prueba estadística es la más adecuada?	A) Prueba U de Mann-Whitney B) Prueba de suma de rangos con signo de Wilcoxon C) t de Student para muestras independientes. D) Kruskal-Wallis. E) Chi-cuadrado	A researcher analyses blood cortisol levels (mg/dL) in 190 patients with diabetes and without diabetes. The data follow a normal distribution, and variances are homogeneous. Which statistical test is the most appropriate?	A) Mann-Whitney U test. B) Wilcoxon signed-rank test. C) Student's t-test for independent samples. D) Kruskal-Wallis. E) Chi-square
3	Se comparan los niveles de ansiedad (leve, moderada y severA) antes y después de una intervención psicológica en un grupo de 15 estudiantes. ¿Qué prueba debe utilizarse?	A) t de Student de muestras emparejadas. B) ANOVA de medidas repetidas. C) Prueba de suma de rangos con signo de Wilcoxon. D) Mann-Whitney. E) Chi-cuadrado	Anxiety levels (mild, moderate and severe) are compared before and after a psychological intervention in a group of 15 students. Which test should be used?	A) Paired Student's t-test. B) Repeated measures ANOVA. C) Wilcoxon signed-rank test. D) Mann-Whitney E) Chi-square
4	Se evalúa la asociación entre sexo y presencia de ansiedad (sí/no) en una muestra de 70 estudiantes de medicina. ¿Cuál es la prueba estadística adecuada?	A) t de Student de muestras independientes. B) ANOVA de una vía. C) Prueba U de Mann-Whitney. D) Chi-cuadrado de Pearson. E) McNemar	The association between sex and the presence of anxiety (yes/no) is evaluated in a sample of 70 medical students. Which statistical test is appropriate?	A) Student's t-test for independent samples. B) One-way ANOVA. C) Mann-Whitney U test. D) Pearson's chi-square. E) McNemar test
5	Un estudio compara el IMC (kg/m²) entre pacientes con dieta hipocalórica y dieta mediterránea. El IMC sigue una distribución normal y la prueba de Levene arrojó un p= 0,001. ¿Cuál es la prueba más adecuada?	A) U de Mann-Whitney. B) Prueba de suma de rangos con signo de Wilcoxon. C) t de Student para muestras independientes. D) Kruskal-Wallis. E) Test de Welch	A study compares BMI (kg/m²) between patients on a hypocaloric diet and a Mediterranean diet. BMI follows a normal distribution, and Levene's test shows p = 0.001. Which test is most appropriate?	A) Mann-Whitney U test. B) Wilcoxon signed-rank test. C) Student's t-test for independent samples. D) Kruskal-Wallis. E) Welch's test
6	Se comparan las puntuaciones de depresión en 130 estudiantes de primer, tercer y sexto año. La prueba de Kolmogorov-Smirnoff arrojó un p=0,001. ¿Qué prueba debe emplearse?	A) ANOVA de una vía. B) t de Student de muestras independientes. C) U de Mann-Whitney. D) Kruskal-Wallis. E) Chi-cuadrado de Pearson	Depression scores are compared among 130 first-, third- and sixth-year students. The Kolmogorov-Smirnov test shows p = 0.001. Which test should be used?	A) One-way ANOVA. B) Student's t-test for independent samples. C) Mann-Whitney U test. D) Kruskal-Wallis. E) Pearson's chi-square
7	Se mide la presión arterial de 100 pacientes antes del tratamiento antihipertensivo y a los tres meses. La prueba de Kolmogorov-Smirnov arrojó p = 0,2. ¿Qué prueba debe emplearse?	A) U de Mann-Whitney. B) Prueba de suma de rangos con signo de Wilcoxon. C) t de Student para muestras dependientes. D) ANOVA de una vía. E) Chi-cuadrado	Blood pressure is measured in 100 patients before antihypertensive treatment and after three months. The Kolmogorov-Smirnov test shows p = 0.2. Which test should be used?	A) Mann-Whitney U test. B) Wilcoxon signed-rank test. C) Paired Student's t-test. D) One-way ANOVA. E) Chi-square
8	Un ensayo clínico compara la glucemia en 16 pacientes antes, a las 4 semanas y a las 12 semanas tras un fármaco A. ¿Cuál es la prueba correcta?	A) t de Student de muestras emparejadas. B) U de Mann-Whitney. C) ANOVA de medidas repetidas. D) ANOVA de una vía. E) Test de Friedman	A clinical trial compares blood glucose levels in 16 patients before, at four weeks and at 12 weeks after drug A. Which is the correct test?	A) Paired Student's t-test. B) Mann-Whitney U test. C) Repeated measures ANOVA. D) One-way ANOVA. E) Friedman test
9	En un estudio piloto, 12 pacientes califican su dolor antes y después de acupuntura en leve, moderado y severo, observándose solo si mejora o empeora. ¿Qué prueba es adecuada?	A) Chi-cuadrado de Pearson. B) Prueba de los signos. C) t de Student para muestras independientes. D) Test de McNemar. E) ANOVA de medidas repetidas	In a pilot study, 12 patients rate their pain before and after acupuncture as mild, moderate or severe, observing only whether it improves or worsens. Which test is appropriate?	A) Pearson's chi-square. B) Sign test. C) Student's t-test for independent samples. D) McNemar test. E) Repeated measures ANOVA
10	Un estudio evalúa la efectividad de una intervención educativa para mejorar la adherencia al tratamiento en 40 pacientes (sí/no). ¿Cuál es la prueba más adecuada?	A) Chi-cuadrado de Pearson. B) t de Student para muestras emparejadas. C) Test de McNemar. D) Prueba de suma de rangos con signo de Wilcoxon. E) ANOVA de una vía	A study evaluates the effectiveness of an educational intervention to improve treatment adherence in 40 patients (yes/no). Which test is most appropriate?	A) Pearson's chi-square. B) Paired Student's t-test. C) McNemar test. D) Wilcoxon signed-rank test. E) One-way ANOVA
11	Se comparan los valores medios de presión arterial sistólica (mmHg) antes y después de iniciar un tratamiento antihipertensivo en un grupo de 100 pacientes. La prueba de Shapiro-Wilk muestra p = 0,32. ¿Qué prueba estadística es la más adecuada?	A) t de Student para muestras independientes. B) t de Student para datos emparejados. C) Test de Welch. D) U de Mann-Whitney. E) ANOVA de una vía	Mean systolic blood pressure values (mmHg) are compared before and after starting antihypertensive treatment in a group of 100 patients. The Shapiro-Wilk test shows p = 0.32. Which statistical test is most appropriate?	A) Student's t-test for independent samples. B) Paired Student's t-test. C) Welch's test. D) Mann-Whitney U test. E) One-way ANOVA
12	Se comparan los niveles medios de hemoglobina (mg/dL) entre 380 personas con diabetes y 180 personas hipertensas. La prueba de Kolmogorov-Smirnov muestra p = 0,21 y la prueba de Levene p = 0,002 en la variable continua. ¿Qué prueba debe utilizarse?	A) t de Student para muestras independientes. B) t de Student para datos emparejados. C) Test de Welch. D) U de Mann-Whitney. E) Prueba de los signos	Mean haemoglobin levels (mg/dL) are compared between 380 people with diabetes and 180 people with hypertension. The Kolmogorov-Smirnov test shows p = 0.21 and Levene's test p = 0.002. Which test should be used?	A) Student's t-test for independent samples. B) Paired Student's t-test. C) Welch's test. D) Mann-Whitney U test. E) Sign test
13	Se desea comparar la cantidad del número de días de hospitalización entre 39 pacientes del servicio de urología y 31 del servicio de nefrología. La prueba de Shapiro-Wilk muestra p = 0,0000001 en ambos grupos. ¿Cuál es la prueba más adecuada?	A) t de Student para muestras independientes. B) t de Student para datos emparejados. C) U de Mann-Whitney. D) Prueba de Wilcoxon para muestras emparejadas. E) ANOVA	The number of hospitalisation days is compared between 39 patients from the urology service and 31 from the nephrology service. The Shapiro-Wilk test shows p = 0.0000001 in both groups. Which test is most appropriate?	A) Student's t-test for independent samples. B) Paired Student's t-test. C) Mann-Whitney U test. D) Wilcoxon signed-rank test for paired samples. E) ANOVA
14	En un estudio se comparan los niveles de colesterol LDL en 890 personas casadas, viudas y divorciadas. La prueba de Kolmogorov-Smirnov muestra p = 0,18 y la prueba de Levene p = 0,42 en la variable continua. ¿Qué prueba estadística debe utilizarse?	A) t de Student. B) ANOVA de un factor. C) Test de Kruskal-Wallis. D) U de Mann-Whitney. E) Test de Friedman	LDL cholesterol levels are compared among 890 married, widowed and divorced individuals. The Kolmogorov-Smirnov test shows p = 0.18 and Levene's test p = 0.42. Which statistical test should be used?	A) Student's t-test. B) One-way ANOVA. C) Kruskal-Wallis test. D) Mann-Whitney U test. E) Friedman test
15	Se comparan los valores de frecuencia cardíaca antes y después de una intervención en 29 pacientes. La prueba de Shapiro-Wilk muestra p = 0,01. ¿Qué prueba es la más adecuada?	A) t de Student para datos emparejados. B) t de Student para muestras independientes. C) U de Mann-Whitney. D) Prueba de suma de rangos con signo de Wilcoxon. E) Test de Welch	Heart rate values are compared before and after an intervention in 29 patients. The Shapiro-Wilk test shows p = 0.01. Which test is most appropriate?	A) Paired Student's t-test. B) Student's t-test for independent samples. C) Mann-Whitney U test. D) Wilcoxon signed-rank test. E) Welch's test
16	Se desea comparar las medias de IMC entre hombres y mujeres. La prueba de Kolmogorov-Smirnov muestra p = 0,27 y la prueba de Levene p = 0,03. ¿Qué prueba estadística debe utilizarse?	A) t de Student para muestras independientes. B) t de Student para datos emparejados. C) Test de Welch. D) U de Mann-Whitney. E) ANOVA	Mean BMI values are compared between men and women. The Kolmogorov-Smirnov test shows p = 0.27 and Levene's test p = 0.03. Which statistical test should be used?	A) Student's t-test for independent samples. B) Paired Student's t-test. C) Welch's test. D) Mann-Whitney U test. E) ANOVA
17	Se comparan los valores medios de colesterol total en tres grupos independientes de pacientes según el tipo de dieta. La prueba de Shapiro-Wilk muestra p = 0,45 en los tres grupos y la prueba de Levene p = 0,62. ¿Qué prueba estadística es la más adecuada?	A) t de Student. B) ANOVA de una vía. C) Kruskal-Wallis. D) Friedman. E) U de Mann-Whitney	Mean total cholesterol values are compared among three independent groups of patients according to diet type. The Shapiro-Wilk test shows p = 0.45 in all groups and Levene's test p = 0.62. Which test is most appropriate?	A) Student's t-test. B) One-way ANOVA. C) Kruskal-Wallis test. D) Friedman test. E) Mann-Whitney U test
18	Se comparan los niveles de triglicéridos (mg/dL) en pacientes de los 10 servicios de un hospital. La prueba de Kolmogorov-Smirnov muestra p < 0,001 en la variable continua. ¿Qué prueba debe utilizarse?	A) ANOVA de una vía. B) t de Student de muestras independientes. C) Kruskal-Wallis. D) Friedman. E) U de Mann-Whitney	Triglyceride levels (mg/dL) are compared among patients from 10 hospital departments. The Kolmogorov-Smirnov test shows p < 0.001. Which test should be used?	A) One-way ANOVA. B) Student's t-test for independent samples. C) Kruskal-Wallis test. D) Friedman test. E) Mann-Whitney U test
19	En un estudio se comparan los valores de glucemia (mg/dL) en tres momentos distintos (basal, 3 meses y 6 meses) en el mismo grupo de pacientes. La prueba de Shapiro-Wilk muestra p = 0,01. ¿Cuál es la prueba más adecuada?	A) ANOVA de una vía. B) ANOVA de medidas repetidas. C) Kruskal-Wallis. D) Friedman. E) U de Mann-Whitney	Blood glucose values (mg/dL) are compared at three different time points (baseline, three months and six months) in the same group of patients. The Shapiro-Wilk test shows p = 0.01. Which test is most appropriate?	A) One-way ANOVA. B) Repeated measures ANOVA. C) Kruskal-Wallis test. D) Friedman test. E) Mann-Whitney U test
20	Se analizan los valores de presión arterial (mmHg) en 30 pacientes que recibieron 3 dosis de losartán (25 mg, 50 mg y 100 mg) durante 3 meses. La distribución no es normal según Kolmogorov-Smirnov p = 0,02. ¿Qué prueba estadística debe utilizarse?	A) ANOVA de una vía. B) Kruskal-Wallis. C) Friedman. D) U de Mann-Whitney. E) t de Student de muestras emparejadas	Blood pressure values (mmHg) are analysed in 30 patients who received three doses of losartan (25 mg, 50 mg and 100 mg) over three months. The distribution is non-normal according to Kolmogorov-Smirnov p = 0.02. Which statistical test should be used?	A) One-way ANOVA. B) Kruskal-Wallis test. C) Friedman test. D) Mann-Whitney U test. E) Paired Student's t-test
